# Epigenetic Regulation Shapes the Stem Cells State

**DOI:** 10.1155/2016/8143407

**Published:** 2016-01-24

**Authors:** Giuseppina Caretti, Libera Berghella, Aster Juan, Lucia Latella, James Ryall

**Affiliations:** ^1^Department of Biosciences, University of Milan, 20133 Milan, Italy; ^2^IRCCS Fondazione Santa Lucia, 00143 Rome, Italy; ^3^National Institute of Arthritis and Musculoskeletal and Skin Diseases, National Institutes of Health, Bethesda, MD 20892-8022, USA; ^4^Institute of Translational Pharmacology, National Research Council (CNR), 00133 Rome, Italy; ^5^Stem Cell Metabolism & Regenerative Medicine Group, Basic & Clinical Myology Laboratory, The University of Melbourne, Melbourne, VIC 3010, Australia

Pluripotent stem cells are endowed with the dual capacity to self-renew and to differentiate towards all lineages. Genetic and genome-wide studies in different model organisms have provided compelling evidence for the importance of epigenetic factors both in the maintenance of pluripotency and in the establishment of cell lineage commitment, during embryonic differentiation and in regenerative events occurring in postnatal life.

In this special issue, we have collected reviews and reports highlighting the plasticity of the epigenome in embryonic, induced pluripotent and adult stem cells, providing readers with an overview of different molecular mechanisms, spanning from DNA methylation, histone modifications and variants, and regulatory RNAs.

In response to signals from the external niche and/or to intracellular signaling pathways, embryonic and adult stem cells engage epigenetic factors in the transition process towards differentiation. L. Fagnocchi et al. have summarized the current understanding of the cross-talk between extrinsic/intrinsic signaling pathways and epigenetic factors and how they cooperatively regulate the fate of different stem cell lineages.

Together with signaling molecules from the niche, metabolites and cofactors derived from the environment modulate intracellular pathways and the epigenetic response. A. J. Harvey et al. review several examples of metabolites and cofactors, which interface metabolic pathways and epigenetic targets, affecting histone marks and transcription.

DNA methylation, once believed to be an irreversible signature restricted to germ cells and embryo development, is now recognized as a dynamic modification, occurring in all cell types. R. C. Laker and J. G. Ryall present recent advances in our knowledge of the role of DNA methylation and hydroxymethylation in skeletal muscle stem cells, with an emphasis on recent whole genome sequencing results that show genomic enrichment for these modifications outside promoter regions and underscore their plastic role in sensing environmental cues.

Recently, the novel function of long noncoding RNAs (lncRNAs) in maintaining pluripotency of ESCs has been explored. A. Rosa and M. Ballarino present an overview of the underlying molecular mechanisms of lncRNAs in regulating ESC pluripotency and differentiation. Another class of noncoding RNAs are presented in the review by A. D. Haase, in which PIWI-interacting RNAs (piRNAs) are described. piRNAs developed transcription and posttranscription strategies to limit the spread of transposon elements, which are mobile genetic elements threatening genomic integrity. The author describes piRNAs as an RNA-based immune system guarding the genome integrity through non-self-memory and adaptive protection against transposons.

Adult stem cells hold great promise for their clinical relevance in regenerative medicine.

In the article by S. Consalvi et al., the authors describe many of the epigenetic regulators involved in the differentiation of skeletal muscle stem cells. The authors focus predominantly on the processes of histone acetylation and deacetylation but also describe a potentially novel role for noncoding RNAs in the epigenetic regulation of differentiation and the potential for epigenetic modulation of skeletal muscle stem cells for the treatment of Duchenne muscular dystrophy (DMD).

In the review by F. A. Choudry and M. Frontini, the authors give an overview on the changes of the epigenetic landscape within the haematopoietic stem cell (HSC) compartment occurring in the elderly, which may be linked to increased occurrence of myeloproliferative disorders, myeloid malignancy, and thrombosis observed in the elderly. Epigenetic changes in the HSC compartment affect HSC activity, survival, and function and they might lead to the selection and expansion of particular HSC clones generating myeloid and platelet skewing of the haematopoietic system distinctive of the elderly population.

The review by L. Rouhana and J. Tasaki focuses on the process of tissue regeneration in lower order organisms. The authors discuss the careful integration of DNA methylation, histone modifications, and noncoding RNAs in the regulation of regeneration, as well as the important role of programmed cell death.

In contrast to changes to the DNA sequence, epigenetic modifications are reversible and are therefore considered promising therapeutic targets for the use of stem cells in the treatment of human diseases. In their review, R. Fernández-Santiago and M. Ezquerra describe how induced pluripotent stem cells are becoming a valuable model for neurodegenerative disorders, recapitulating key disease-associated molecular events. Furthermore, these authors highlight the potential of epigenetic regulation of patient-specific iPSC-derived neural models to develop novel therapeutic approaches for human disorders.

During the cellular reprogramming of somatic cells, distinctive chromatin status coupled with gene expression changes is an important determinant for the reprogramming efficiency towards pluripotency. In the research paper contributed by F. Dong et al., the authors showed that redistribution of histone variants H2A.Z during the reprogramming process alters nucleosome stability to increase expression of genes that promote reprogramming.

Together with kinase inhibitors, cocktails of epigenetic modulators can be used to promote reprogramming and to probe stem cells functions. In their report, Y.-C. Han et al. describe a novel method to induce neuronal stem cells from mouse embryonic fibroblasts, with the use of small molecules, and suggest that the reprogramming is enhanced by histone demethylation and histone acetylation and decreased DNA methylation.

Transdifferentiation is an alternative approach to somatic reprogramming of induced pluripotent stem cells, which allows the direct conversion of one cell type into another, bypassing safety concerns related to the pluripotent cell state. G. Palazzolo and colleagues present an original research paper documenting a transdifferentiation process used to convert fibroblasts from golden retriever dogs with muscular dystrophy (GRMD) directly to cardiac-like myocytes. While the induced cells do not exhibit spontaneous contraction* in vitro*, when transplanted into the hearts of neonatal mice, the induced cells were found to participate in cardiac myogenesis.

Overall, this special issue highlights recent advances in our understanding of epigenetic regulation of stem cells and describes several new approaches to investigate stem cell biology to model human disorders and develop novel therapies for disease states.

*Giuseppina Caretti* *Giuseppina Caretti* *Libera Berghella* *Libera Berghella* *Aster Juan* *Aster Juan* *Lucia Latella* *Lucia Latella* *James Ryall* *James Ryall*

